# Anticancer Effect of *Citrus hystrix* DC. Leaf Extract and Its Bioactive Constituents Citronellol and, Citronellal on the Triple Negative Breast Cancer MDA-MB-231 Cell Line

**DOI:** 10.3390/ph13120476

**Published:** 2020-12-18

**Authors:** Yathsoeung Ho, Nungruthai Suphrom, Krai Daowtak, Pachuen Potup, Yordhathai Thongsri, Kanchana Usuwanthim

**Affiliations:** 1 Cellular and Molecular Immunology Research Unit, Faculty of Allied Health Sciences, Naresuan University, Phitsanulok 65000, Thailand; yathsoeung@gmail.com (Y.H.); kraid@nu.ac.th (K.D.); pachuenp@nu.ac.th (P.P.); yordhathait@nu.ac.th (Y.T.); 2 Department of Chemistry, Faculty of Science and Center of Excellence for Innovation in Chemistry, Naresuan University, Phitsanulok 65000, Thailand; suphrom.n1@gmail.com

**Keywords:** triple negative breast cancer, MDA-MD-231, *Citrus hystrix* DC., leaf extract, citronellol, citronellal

## Abstract

Triple negative breast cancer is one of the most aggressive breast cancer type with abilities of early metastasis and chemoresistance. The tropical plant *Citrus hystrix* DC. has been reported to promote many biological activities including anticancer. However, the effect of *C. hystrix* against triple negative breast cancer has not yet been identified. This study aimed to evaluate the anticancer properties of *C. hystrix* leaf extract and its bioactive constituents citronellol and citronellal against the triple negative breast cancer MDA-MB-231 cell line. *C. hystrix* leaves were powdered and sequentially macerated. The in vitro anticancer effects of *C. hystrix* leaf extracts, and its bioactive constituents (citronellol and citronellal) were evaluated against MDA-MB-231 cell line using cytotoxic MTT assay, cell proliferation, wound scratch migration, colony formation, cell cycle, apoptosis assay, Hoechst staining, RT-qPCR, and Western blot analysis. Results showed that crude hexane extract, citronellol, and citronellal significantly reduced cell proliferation, colony formation, and cell migration by inducing cell cycle arrest, while also inducing apoptosis in MDA-MB-231 cells through inhibition of anti-apoptotic Bcl-2 expression, leading to activation of the caspase-3-dependent pathway. This study is the first report to demonstrate the effect of *C. hystrix*, citronellol, and citronellal against triple negative breast cancer MDA-MB-231 cells.

## 1. Introduction

Triple negative breast cancer (TNBC) is a sub-type of breast cancer. TNBC is characterized by tumors that do not express estrogen receptor (ER) or progesterone receptor (PR), with immunohistochemical (IHC) staining present at less than 1% repartitioning of these receptors in the tissue and less than 10% for human epidermal receptor 2 (Her-2) [1,2]. TNBC accounts for 15–20% of invasive breast cancers in women [3] and presents a higher frequency of recurrence and lower overall survival rate compared to non-TNBC patients [4]. It is one of the most aggressive types of breast cancer due to its early metastasis and resistance to chemotherapies such as anthracyclines, taxanes, and many others [5]. Several new anticancer plant-derived molecules have been put through clinical trials [6] and some such as cabazitaxel and homoharringtonine have been approved by the FDA for use in treatment resistance [7,8]. Plant materials are now becoming increasingly recognized as a substantial source of anticancer agents and urgently require assessment for future development.

*Citrus hystrix* DC., also known as kaffir lime, is a tropical plant belonging to the Rutaceae family. The tree can grow up to six meters high and has double pointy oval shaped leaf, white fragrant flowers, and green to yellow ellipsoid fruits with irregular bumpy skin. Many compounds have been identified in the fruit including phenolics, flavonoids, terpenoids, alkaloids, coumarins, glycosides, saponins, tannins, hydrocarbons, and fatty acids [9,10,11]. In Thailand, *C. hystrix* is believed to have cancer-prevention and anti-inflammation properties and it is used as a remedy in traditional medicine [12]. *C. hystrix* has also been reported to exert potency against many types of cancers. It induced selective cytotoxicity against brain and cervical cancer cells [13], induced cell cycle arrest in lymphoma cells [14], and also suppressed cell migration and induced cell shrinkage in human pancreatic cancer cells [15]. Citronellol and citronellal are two acyclic monoterpenoids that have been identified in *C. hystrix*. They have been reported to exert many varied biological activities including anti-inflammatory and anti-microbial activities [16]. Citronellal exhibited toxicity against MCF-7 breast cancer cells [17] and reduced proliferation by inducing apoptosis in Huh7 hepatocellular carcinoma [18], while citronellol induced necroptosis of lung cancer [19]. Both molecules have also been reported to inhibit the action of multidrug resistance P-glycoprotein, which ameliorates the efficacity of drug treatments [20]. However, the effect of *C. hystrix* and its bioactive compounds citronellol and citronellal have not yet been observed on triple negative breast cancer. Here, the anticancer effects of *C. hystrix* crude extracts, citronellol, and citronellal were evaluated against the triple negative breast cancer MDA-MB-231 cell line in terms of cell proliferation, migration, cell cycle arrest and apoptosis. Findings revealed that *C. hystrix* leaf extract and its active compounds citronellol and citronellal strongly inhibited tumor cell growth and triggered apoptosis via over-expression of cleaved caspase-3 and Bax, while down-regulating the anti-apoptotic Bcl-2.

## 2. Results

### 2.1. Extraction Yields of C. hystrix Leaf Powder

Maceration *C. hystrix* leaf powder with hexane solvent resulted in a sticky dark green extract with yield of 1.1% (*w/w* dried powder). The following sub-sequential extractions culminated in a similar consistency of extracts with yield of 1.8% obtained using ethyl acetate and 7.6% using 95% ethanol.

### 2.2. Identification of Volatile Components in Crude Hexane Extract by GC-MS

Analysis of the total ionic chromatogram by GC-MS identified 45 volatile compounds from crude hexane extract. Relative amounts (%) of the compositions were calculated by peak-area normalization (Table 1). Most of the identified compounds of crude *C. hystrix* hexane extract were terpenoids (19.84%). The extract contained oxygenated monoterpenes (3.99%), hydrocarbon monoterpenes (2.19%), oxygenated sesquiterpenes (7.67%), and hydrocarbon sesquiterpenes (5.99%) as major compounds. The identification of two oxygenated monoterpenes, citronellal and citronellol, were found in leaf extract and then confirmed with authentic standards which they were observed at retention time (RT) at 10.75 min and 12.85 min, respectively (Figure S1). Other constituents comprised long-chain hydrocarbons, phytosterols, fatty acids, fatty alcohols, and vitamins.

### 2.3. Cytotoxicity of Crude Extracts, Citronellol, and Citronellal

**Figure 1:**
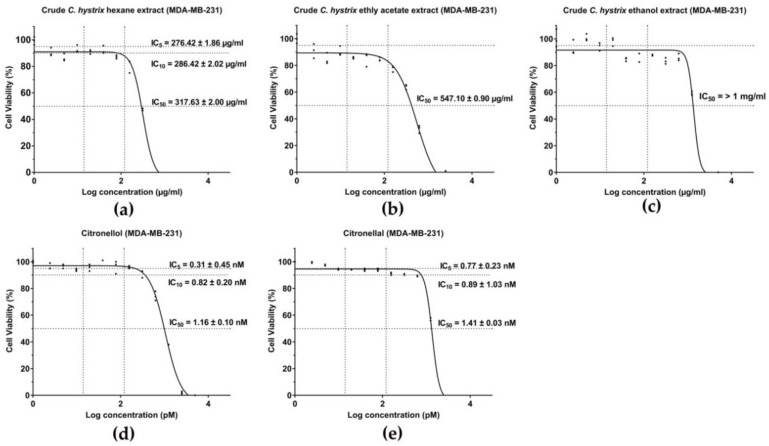
IC_50_ value of treatments on MDA-MB-231 cells: (**a**) Crude hexane extract, (**b**) crude ethyl acetate extract, (**c**) crude ethanolic extract, (**d**) citronellol, and (**e**) citronellal.

After 24 h of treatment, half maximal inhibitory concentration (IC_50_) values were obtained using a dose-response inhibition curve. *C. hystrix* crude extracts, citronellol, and citronellal reduced cell viability of MDA-MB-231 cells with IC_50_ of 317.63 ± 2.00 µg/mL for crude hexane extract (Figure 1a), 547.10 ± 0.90 µg/mL for crude ethyl acetate extract (Figure 1b) and IC_50_ > 1000 µg/mL for crude ethanolic extract (Figure 1c). For citronellol, IC_50_ values were 1.16 ± 0.10 nM (Figure 1d) and 1.41 ± 0.03 nM for citronellal (Figure 1e). The cytotoxicity of crude hexane extract, citronellol, and citronellal on normal cells, human monocyte-derived macrophages, were performed as the experimental model (Figure S2).

### 2.4. Effect of Crude Hexane, Citronellol, and Citronellal on Cell Proliferation

**Figure 2:**
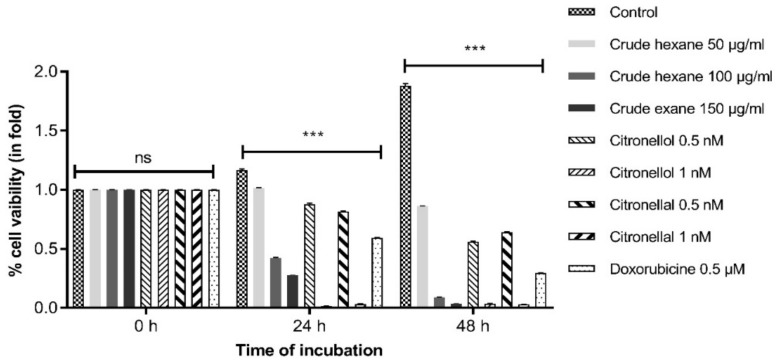
Crude hexane extract, citronellol, and citronellal reduced MDA-MB-231 cell proliferation at both 24 h and 48 h. Data are presented as means ± SEM. One-way ANOVA with Dunnett’s multiple comparisons test was used to analyze the difference between control and treatment groups, *p* value < 0.05 considered significant (*** *p* < 0.001).

The cytotoxicity of crude hexane extract, citronellol and citronellal on cell proliferation rate of MDA-MB-231 cells was performed by the MTT assay. For the non-treated group, cell viability value doubled at 48 h, indicating that the cells significantly duplicated their population. By contrast, results showed that all treatment groups significantly reduced proliferation of MDA-MB-231 cells in both a dose- and time-dependent manner when compared to the non-treated group at each specific incubation time of 24 h and 48 h (Figure 2).

### 2.5. Crude Hexane, Citronellol, and Citronellal on Inhibited MDA-MB-231 Cell Migration

**Figure 3:**
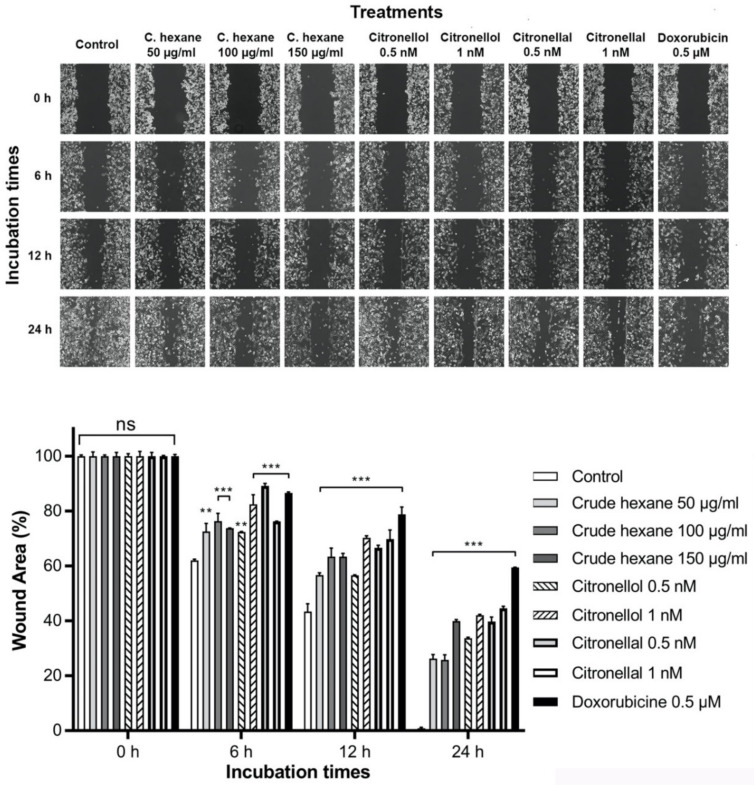
Cells migration into wound areas was observed under microscope at 6 h, 12 h, and 24 h. ImageJ 1.52a software was used to analyze cells migration into wound areas. Data are presented as means ± SEM. One-way ANOVA with Dunnett’s multiple comparisons test was used to analyze the difference between control and treatment groups, *p* value < 0.05 considered significant (** *p* < 0.01, *** *p* < 0.001).

Cell migration is one of the important processes in cancer metastasis. The effects of crude hexane extract, citronellol, and citronellal on cell migration were evaluated by in vitro wound scratch migration assay. In the control group, MDA-MB-231 cells migrated to completely close the wound areas at 24 h (Figure 3). However, in the treatment groups, crude hexane extract, citronellol, and citronellal significantly reduced cell migration into wound areas at 6 h, 12 h, and 24 h in both a dose- and time-dependent manner.

### 2.6. C. hystrix Hexane Extract, Citronellol, and Citronellal Reduced Number of Colonies Forming in MDA-MB-231 Cells

**Figure 4:**
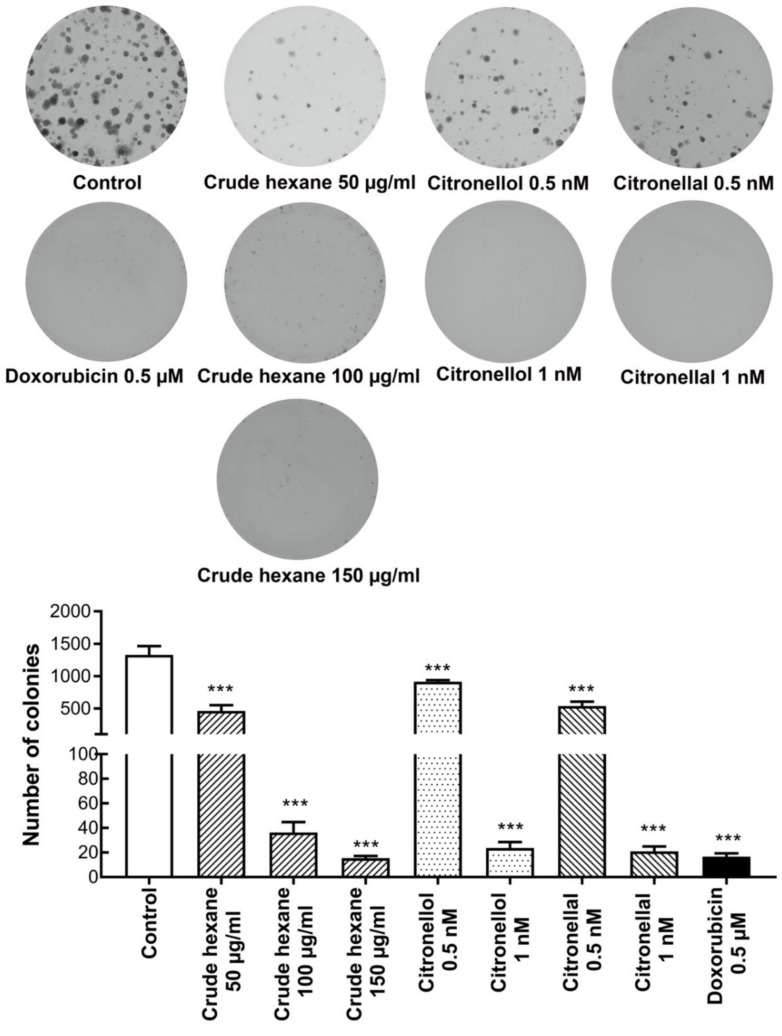
Colonies picture as 8-bit grayscale images under different treatments. Crude hexane, citronellol, and citronellal reduced number of MDA-MB-231 cell colonies in a dose-dependent manner. Data are presented as means ± SEM. One-way ANOVA with Dunnett’s multiple comparisons test was used to analyze the difference between control and treatment groups, *p* value < 0.05 considered significant (*** *p* < 0.001).

The colony forming assay was used to evaluate cell survival by studying cell growth from a single cell to form colonies in response to toxic substances. Here, colony formations of MDA-MB-231 cells under treatments by crude hexane extract, citronellol, citronellal, and doxorubicin were observed. In the treatment groups, crude hexane extract, citronellol, and citronellal significantly reduced colony formation of MDA-MB-231 cells in a dose-dependent manner, while only a few colonies were formed under treatment of doxorubicin 0.5 µM (Figure 4).

### 2.7. Crude Hexane, Citronellol, and Citronellal Induced Cell Cycle Arrest in MDA-MB-231 Cell

**Figure 5:**
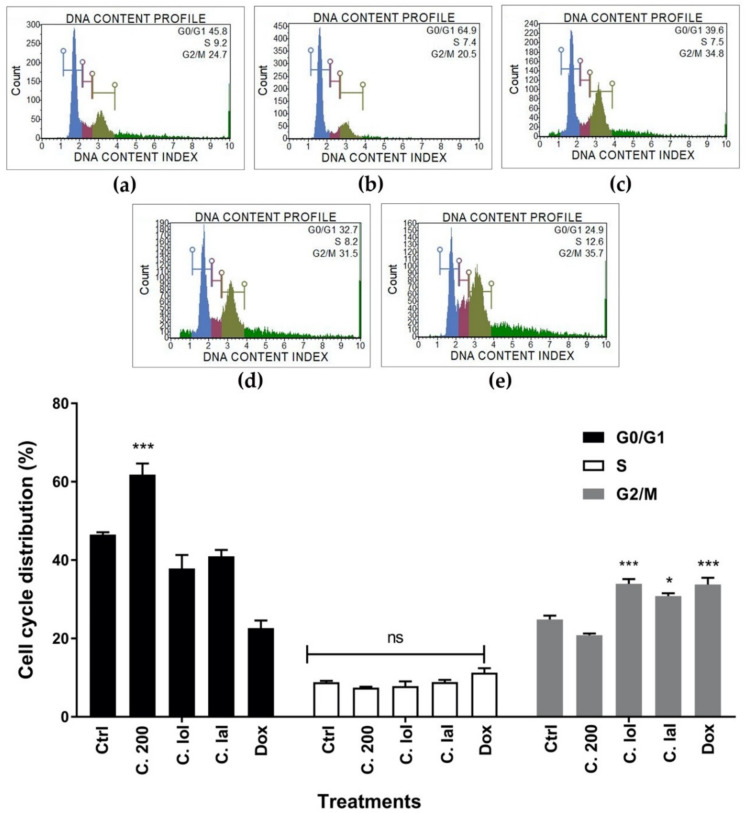
Cell cycle arrest induced by treatment groups on MDA-MB-231 cells. (**a**) Control (Ctrl), (**b**) crude hexane extract 200 µg/mL (C.200), (**c**) citronellol 1 nM (C. lol), (**d**) citronellal 1 nM (C. lal), (**e**) Doxorubicin 0.5 µM (Dox). Data are presented as means ± SEM. One-way ANOVA with Dunnett’s multiple comparisons test was used to analyze the difference between control and treatment groups, *p* value < 0.05 considered significant (*** *p* < 0.001, * *p* < 0.05).

Cell cycle arrest is a mechanism that cells use to prevent cell cycle progression when they encounter a toxic substance, DNA damage during DNA replication. In the treatment groups, crude hexane extract significantly induced cell cycle arrest at the G0/G1 phase when compared to the non-treated group. However, in the citronellol- and citronellal-treated groups, the cells were significantly arrested at the G2/M phase, while doxorubicin significantly induced cell cycle arrest at the G2/M phase (Figure 5).

### 2.8. Crude Hexane, Citronellol, and Citronellal Induced Apoptosis in MDA-MB-231 Cells

**Figure 6:**
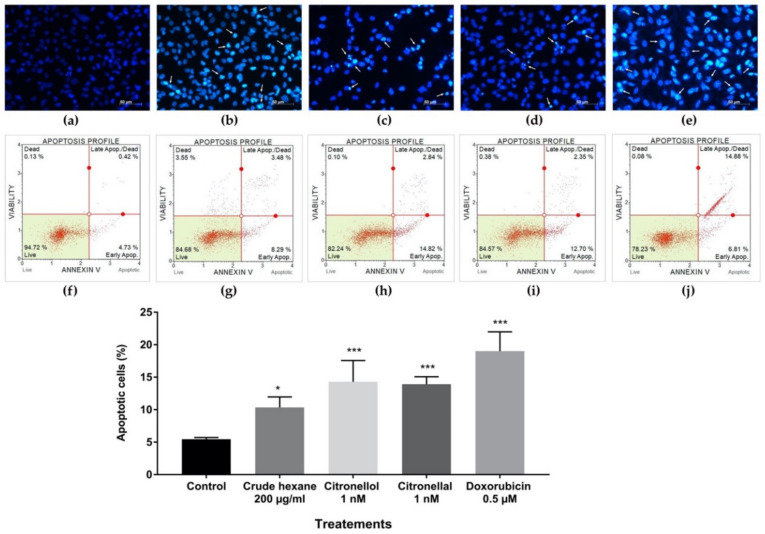
Hoechst 33342 staining showing chromatin condensation and DNA fragmentation in apoptotic cells under treatment of (**a**) control, (**b**) crude hexane extract 200 µg/mL, (**c**) citronellol 1 nM, (**d**) citronellal 1 nM, (**e**) doxorubicin 0.5 µM. Apoptotic MDA-MB-231 cells using Annexin-V and 7-AAD staining under treatment of (**f**) control, (**g**) crude hexane extract 200 µg/mL, (**h**) citronellol 1 nM, (**i**) citronellal 1 nM, (**j**) doxorubicin 0.5 µM. Data are presented as means ± SEM. One-way ANOVA with Dunnett’s multiple comparisons test was used to analyze the difference between control and treatment groups, *p* value < 0.05 considered significant (* *p* < 0.05, *** *p* < 0.001).

Crude hexane extract, citronellol, and citronellal reduced cell proliferation and migration, while also decreasing the number of colony formations of MDA-MB-231 cells. When cells encounter toxic substances, they undergo a repair mechanism that reduces their active state. However, if the damage cannot be repaired, cell death or apoptosis results. Hoechst 33342 staining was used to confirm morphological change during apoptosis. The non-treated group showed normal nuclear cell structure; however, in the treatment groups, some condensed blue fluorescence appeared in cell nuclei indicating chromatin condensation and/or DNA fragmentation in cells treated by crude hexane extract 200 µg/mL (Figure 6b), citronellol (Figure 6c), citronellal (Figure 6d), and doxorubicin 0.5 µM (Figure 6e). Furthermore, apoptotic cells under treatments of crude hexane extract, citronellol, and citronellal for 24 h were observed using the Muse^TM^ Cell Analyser. Results revealed that a dose of 200 µg/mL crude hexane extract induced apoptosis in MDA-MB-231 cells at up to 11.02 ± 0.53% (Figure 6g) when compared to the control (Figure 6f). Similarly, citronellol at 1 nM induced cell apoptosis of 14.30 ± 0.89% (Figure 6h) with 13.92 ± 0.31% for citronellal at 1 nM (Figure 6i), while doxorubicin at 0.5 µM induced cell apoptosis of 19.02 ± 0.81% (Figure 6j).

### 2.9. Crude Hexane, Citronellol, and Citronellal Modulated Apoptosis-Related Proteins Gene Expression in MDA-MB-231 Cells

**Figure 7:**
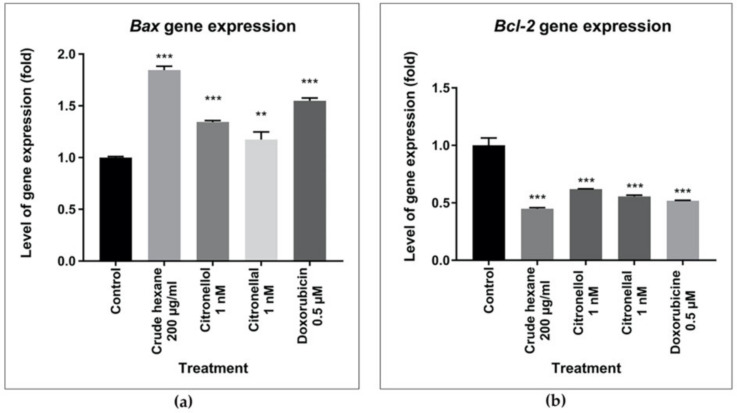
Alteration apoptosis-related genes expression (**a**) *Bax* and (**b**) *Bcl-2*. Data are presented as means ± SEM. One-way ANOVA was used to analyze the difference between control group and treatment groups. *p* value < 0.05 considered significant (** *p* < 0.01, *** *p* < 0.001).

The expression level of apoptosis-related genes was performed using RT-qPCR to better understand cell apoptosis. Cells were treated 24 h before mRNA extraction. Results showed that the expression level of the *Bax* gene significantly increased in the treatment of crude hexane extract 200 µg/mL, citronellol 1 nM, citronellal 1 nM and doxorubicin 0.5 µM (Figure 7a). On the other hand, expression level of the *Bcl-2* gene was markedly downregulated in each treated group compared to the control (Figure 7b).

### 2.10. Crude Hexane, Citronellol, and Citronellal Induced Apoptosis and DNA Fragmentation in the Cells by Inhibiting the Anti-Apoptotic Bcl-2 Protein and Activating Caspase Dependent Apoptotic Pathway

**Figure 8:**
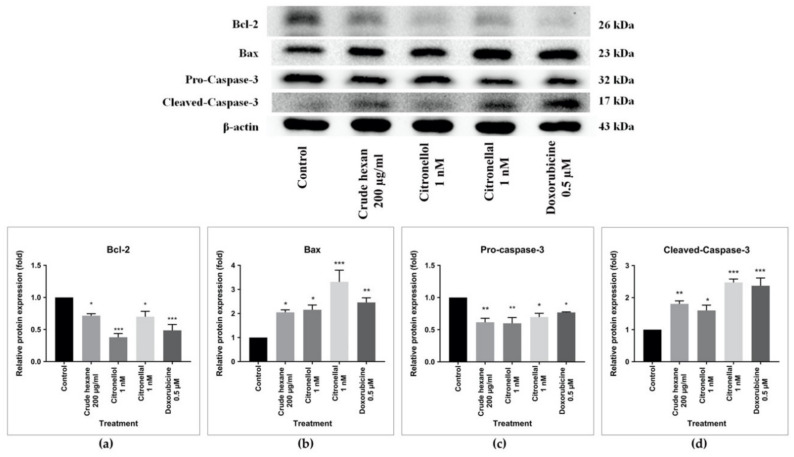
Western Blot analysis on: (**a**) Bcl-2 protein, (**b**) Bax protein, (**c**) Pro-Caspase-3, (**d**) Cleaved-Caspase-3. Full length of Western blot analysis (Figure S3). Data are expressed as mean ± SEM. One-way ANOVA was used to analyze the difference between control group and treatment groups, *p* value < 0.05 considered significant (* *p* < 0.05, ** *p* < 0.01, *** *p* < 0.001).

To further confirm the results, another experiment was conducted to measure the protein expression of both Bax and Bcl-2 proteins using Western blot analysis. Surprisingly, the expression of Bcl-2 protein significantly decreased after treatment by crude hexane extract 200 µg/mL, citronellol 1 nM, citronellal 1 nM, and doxorubicin 0.5 µM compared to the control group (Figure 8a), while Bax protein expression was upregulated in the same treated groups (Figure 8b). In response to the treatments, cells upregulated Bax protein expression and downregulated Bcl-2 protein expression. Alteration of Bax/Bcl-2 expression may lead to the activation of caspase-3 protein, which is an executioner in apoptosis caspase-dependent activation. To confirm this hypothesis, pro-caspase-3 and cleaved-caspase-3 protein levels were measured by Western blot analysis. Results demonstrated a significant decrease in intensities of pro-caspase-3 in the treatment groups compared to the control (Figure 8c), with significantly increase cleaved-caspase-3 protein intensity (Figure 8d).

## 3. Discussion

*C. hystrix* has been reported to possess many biological activities. In this experiment, we extracted *C. hystrix* leaf by the sequential maceration method using lower polar solvent to higher polar solvent (hexane, ethyl acetate, and 95% ethanol). Higher yield was obtained from the ethanolic extract compared to crude hexane and ethyl acetate extract. The MTT assay showed that crude *C. hystrix* hexane extract had higher efficiency in reducing MDA-MB-231 cell viability compared to the two other crude extracts, while the two pure compounds showed similar toxicity on MDA-MB-231 cell viability. Moreover, TLC fingerprints of crude *C. hystrix* hexane extract showed more trace of compounds compared to other two extracts with the trace of citronellol and citronellal could be identified at similar retention factor (R_f_) (Figure S4). A previous study reported that crude ethyl acetate and crude ethanolic extract showed a higher effect against HeLa cells compared to crude hexane extract [21]. Doxorubicin is an anticancer agent that is widely used in cancer treatments. It acts as an anti-topoisomerase 2, which prevents DNA from replication [22]. We used doxorubicin as a drug control in all experiments.

Crude *C. hystrix* hexane extract was selected for chemical constituent analysis by GC-MS. Results indicated that terpenoids comprised the most compounds identified from *C. hystrix* leaf extract, while citronellal (0.67%) and citronellol (1.42%) of *C. hystrix* leaf crude hexane extract from the GC chromatogram appeared at retention times of 10.75 min and 12.85 min, respectively.

We then tested the effect of crude hexane extract, citronellol, and citronellal on the proliferation of MDA-MB-231 breast cancer cells. Results showed that crude hexane extract significantly reduced cell growth rate compared to the non-treated group. Anti-proliferative effects of *C. hystrix* were also reported in previous studies on leukemic cell lines [23] and on HeLa cervical cancer cells [21], while the anti-proliferative effect on MDA-MB-231 cells was also significant under treatments of both citronellol and citronellal. A previous study reported that citronellal reduced proliferation in Huh7 hepatocellular carcinoma cells [18], while citronellol inhibited non-small cell lung carcinoma A-549 cells [24]. Further experiments were performed to support the results of pre-existing anti-proliferative effects of both crude hexane extract and the two pure compounds. Clonogenic assay was performed to mimic cancer growth from single mutated cells to form cancer cell colonies or tumor mass. In the control group, MDA-MB-231 cells grew and formed cell colonies within 15 days of incubation. However, in the treatment groups, crude hexane extract, citronellol, and citronellal significantly reduced the number of colonies and inhibited colony formation at higher doses. Bergamottin was also found to inhibit colonies forming in PANC-1 human pancreas cancer [15]. Surviving under a tough environment with highly proliferative and migrative abilities are keys for successful cancer development or metastasis. Therefore, decreasing these two factors could be a suitable strategy to prevent early metastasis of the cancer. To test this hypothesis, wound scratch migration assay was conducted to study the effects of crude hexane extract, citronellol, and citronellal on cell migration. Results indicated that the treatments groups slowed down cell migration into wound areas in a dose and time dependent manner. Cell cycle analysis and apoptosis assay were performed to better understand whether the anti-proliferative and anti-migrative effects of crude hexane extract, citronellol, and citronellal were linked to these events. Results showed that a significant number of MDA-MB-231 cancer cells were arrested at the G0/G1 phase by *C. hystrix* crude hexane extract. Cells treated with citronellol and citronellal accumulated at the G2/M phase in the same way as treatment by doxorubicin. However, *C. hystrix* leaf extract showed different effects on the Molt-4 cell line by inducing cell cycle arrest at the G2/M phase [14]. Citronellol was also reported to induce lung cancer NCI-H1299 cell cycle arrest at the G0/G1 phase [19].

Results indicated a significant number of apoptotic cells under treatments of crude hexane extract, citronellol, and citronellal, while morphological changes as chromatin condensation and swelling were seen under the treatment groups. To help define a support mechanism for cell apoptosis, the levels of genes and proteins involved were analyzed using RT-qPCR and Western blot techniques. Results proved that levels of the *Bcl-2* gene and protein expression decreased under the treatment group, while the *Bax* gene and protein expression increased in intensity. During apoptosis, cleavage of DNA by endonuclease occurs after the activation of caspase-3 [25]. Results from Western blot analysis clearly demonstrated the activation of caspase-3 through decreasing density of total caspase-3 in the treated groups compared to the control and increasing the intensity of cleaved-caspase-3 protein, indicating activation of the caspase-dependent apoptosis pathway. The induction of apoptosis in MDA-MB-231 cells by the treated groups was probably activated by the caspase-dependent apoptosis pathway through inhibition of anti-apoptotic protein Bcl-2, leading to an increase of pro-apoptotic Bax protein activity. Bax protein mediated the pore formation complex at the outer membrane of mitochondria and consequentially triggered the release of cytochrome C, leading to the activation of caspase 3 protein. The effect of doxorubicin inducing apoptosis through the caspase-dependent apoptosis pathway was also reported [22]. Bergamottin, a compound identified in *C. hystrix*, induced apoptosis in human pancreas cancer cells PANC-1 by inhibiting the Akt/mTOR signaling pathway, leading to inhibition of cell survival, proliferation, and migration [15]. However, further research is necessary to better understand the mechanism behind cell cycle arrest to reduce cell migration. Animal model experiments should also be launched to enhance drug development of *C. hystrix* leaf extract and its bioactive compounds citronellol and citronellal.

## 4. Materials and Methods

### 4.1. Chemicals and Reagents

β-Citronellol, citronellal (analytical standard grade), and doxorubicin were purchased from Sigma-Aldrich, Inc., St. Louis, MO, USA, and stored at 4 °C. Dimethyl sulphoxide (DMSO) was used to dissolve the crude extracts and doxorubicin was dissolved with deionized water.

### 4.2. Plant Materials and Extraction Process

*C. hystrix* leaf fine powder was received from Khaolaor Laboratories Co., Ltd., Nakhon Pathom, Thailand (COA no. 250818) and crude extracts were obtained using the sequential maceration method. Finely powdered leaves (500 mg) were extracted with 1000 mL of hexane and stirred continuously for 3 days at room temperature. The mixture was then filtered and evaporated using a rotary evaporator to give crude *C. hystrix* hexane extract. The maceration procedure was repeated three times. The marc was further extracted with ethyl acetate and 95% ethanol using the same procedure. The filtrates obtained were evaporated and stored at −20 °C until required for use.

### 4.3. Cell Culture

MDA-MB-231 cell line (ATCC^®^ HTB-26™ATCC) was maintained in Dulbecco’s Modified Eagle Medium (DMEM; Gibco™; Thermo Fisher Scientific, Inc., Waltham, MA, USA) with 10% fetal bovine serum (FBS; Gibco™; Thermo Fisher Scientific, Inc., Waltham, MA, USA) and 1% of antibiotic-antimycotic (amphotericin B, penicillin, streptomycin) (Gibco™; Thermo Fisher Scientific, Inc., Waltham, MA, USA) at 37 °C, CO_2_ 5%.

### 4.4. Human Monocyte Isolation

Human monocytes were isolated from fresh buffy coat by Ficoll-Paque™ (Sigma-Aldrich, Inc.) using the density gradient centrifugation method. Human monocyte-derived macrophages were obtained by culturing the freshly isolated monocytes in RPMI-1640 medium (Gibco™; Thermo Fisher Scientific, Inc., Waltham, MA, USA) with 10% FBS incubated at 37 °C, CO_2_ 5% for one week before performing the experiments. Buffy coat was obtained from the Blood Bank, Naresuan University Hospital, Phitsanulok, Thailand. Ethics approval was obtained from the Human Ethics Committee of Naresuan University (IRB no. 0945/62).

### 4.5. Gas Chromatography-Mass Spectrometry Analysis (GC-MS)

The analysis of volatile compounds in crude hexane extract from *C. hystrix* leaf powder was performed using a Hewlett Packard Gas Chromatograph model 6890 (Agilent Technologies, Palo Alto, CA, USA) equipped with a mass selective detector. Crude hexane extract (50 mg/mL) was prepared by dissolving in hexane and then injected into GC-MS system. Volatile compounds in sample were separated using silica capillary Hewlett Packard HP-5 (5% phenyl methyl siloxane) column (30 m × 0.25 mm i.d., 0.25 µm film thickness). High-purity helium was used as the carrier gas with constant flow rate at 13.7 mL/min. Initial injector temperature was set at 250 °C with split ratio mode at ratio 10:1 and 1 µL injector volume. The oven temperature was started at 70 °C for 3 min, then increased to 280 °C (5 °C/min) and held for 20 min. Transfer temperature was set at 280 °C and the mass detection ranges were set from 50 to 700 amu in full scan. Retention indices (RIs) were determined by analyzing a solution containing a homologous series of n-alkanes (C_8_–C_32_) under the same chromatographic conditions and then calculated as described by van Den Dool and Dec. Kratz [26]. Identification of the volatile components was performed by computer matching of their recorded mass spectra fragmentation patterns with those stored in the Wiley 7n MS spectral library. Further identification was made by comparison of their mass spectra and their RIs relative to n-alkanes with those of the National Institute of Standards and Technology (NIST) Chemistry WebBook [27,28] or with previously published data. The presence of citronellol and citronellal in the extract was also confirmed by analyzing authentic standards under the same chromatographic conditions. Relative contents of each component in the sample were also calculated based on the normalization of peak areas as the percentage of total detected volatile components.

### 4.6. Cell Viability MTT Assay

MDA-MB-231 cells were plated in 96-well plates at density of 10^4^ cells/well and 5 × 10^4^ for human monocyte derived macrophages cells/well. Cells were treated by various concentrations of each crude extracts, citronellol, citronellal for 24 h, and 48 h for doxorubicin. All treatments were then removed, and cells were then washed by phosphate saline buffer (PBS). Culture medium was used as vehicle control. 0.5 mg/mL of 3-(4,5-dimethylthiazol-2-yl)-2,5-diphenyl tetrazolium bromide salt (MTT) from Thermo Fischer Scientific (Waltham, MA, USA) was introduced to cells with 3 h of incubation. The MTT was discarded, and the dye was solubilized by 100 µL of DMSO. Absorbances of the dissolved tetrazolium salt was measured at 570 nm by ELISA microplate reader (PerkinElmer, Inc., Waltham, MA, USA). The IC50 value was generated by dose-response curves using GraphPad Prism 7.

### 4.7. Identification Effect of Crude Hexane, Citronellol, and Citronellal on Cell Proliferation

MDA-MB-231 cells were seeded at a density of 2 × 10^4^ cells/well and treated with 50, 100, and 150 µg/mL crude hexane extract, 0.5 and 1 nM of citronellol and citronellal, and 0.5 µM doxorubicin for 24 h and 48 h. Cells treat with culture medium only was used as vehicle control. Cell viability was measured at 24 h and 48 h to represent the growth of the cells.

### 4.8. Clonogenic Assay

Cells were seeded at a density of 500 cells/well in 6-well plates for 24 h. Different concentrations of crude hexane extract, citronellol, citronellal, and doxorubicin were treated on cells for 24 h. Cells treated with culture medium only were used as vehicle control. Treated cells were washed by PBS and then cultured in culture medium for 10 days with medium replacement every 3 days. Cells were washed twice with PBS and fixed with 10% neutral buffer formalin for 30 min before fixing by 0.5% crystal violet for 1 h. Pictures of colonies were captured using a Canon macro lens 50 mm/f1.8 STM. The photos were converted to 8-bit greyscale images. Numbers of colonies of MDA-MB-231 cells were counted by Colony Area plugin using ImageJ 1.52a software.

### 4.9. Wound Scratch Migration Assay

MDA-MB-231 cells were seeded and cultured to confluence. SPLScar™ cell scraper (SPL Life Sciences, Gyeonggi-do, Korea) was used to create wound areas. Cells were washed twice by PBS to remove cell debris and floating cells before treatment with various concentrations of crude hexane extract, citronellol, citronellal, and doxorubicin. Cells treated with culture medium only were used as vehicle control. Cell migration into the wound areas was then observed under an inverted microscope (Zeiss Microscopy, Oberkochen, Germany) with objective 10× magnification. Wound closed areas were quantified by MRI_Wound_Healing_Tool plugin using ImageJ 1.52a software.

### 4.10. Cell Cycle Analysis

A total of 200 µg/mL crude hexane extract, 1 nM citronellol and citronellal, and 0.5 µM doxorubicin were treated with MDA-MB-231 cells for 24 h. Cells treat with culture medium only was used as vehicle control. Cells were harvested, transferred into a 1.5 mL microtube, and washed twice by cold PBS. Cells were then fixed with 1 mL of 70% ethanol for 5 h. Two hundred microliters of cell suspension (10^6^ cells/mL) were washed with 250 µL of PBS and 200 µL of Muse™ cell cycle reagent and then incubated in the dark for 20 min at room temperature. The apoptotic cells were analyzed using a Muse™ cell analyzer (Merck, Darmstadt, Germany).

### 4.11. Apoptosis Analysis

Crude hexane extract, citronellol, citronellal, or doxorubicin were treated on MDA-MB-231 cells for 24 h. Cells treat with culture medium only was used as vehicle control. Cells were harvested and transferred into a 1.5 mL microtube and washed twice in cold PBS. Then, the cells were resuspended in 1% BSA and 100 µL of Muse™ Annexin V and dead cell reagent (Merck, Darmstadt, Germany) were added to 100 µL of cell suspension (5 × 10^5^ cells/mL) and incubated in the dark for 20 min at room temperature. The apoptotic cells were analyzed using Muse™ cell analyzer, gated on Annexin V-FITC positive cells and 7-AAD positive cells.

### 4.12. Hoechst 33342 Staining

Cells were cultured on cover slides for 24 h and then treated with crude hexane extract, citronellol, citronellal, or doxorubicin for 24 h. Cells treated with culture medium only were used as vehicle control. The cells were washed twice with PBS and fixed with 4% formaldehyde for 15 min at room temperature. After washing twice with PBS, the cells were permeabilized with 0.15% Triton-X 100 for 15 min at room temperature, followed by washing twice with PBS. An aliquot of 4 μg/mL of Hoechst 33342 solution was used to stain cells for 10 min in the dark at room temperature. Then, 70% glycerol was used as an anti-fade solution. Cover slides were then mounted on the slides and sealed with nail polisher. DNA fragmented or chromatin condensed cells were observed under a fluorescence microscope (Zeiss Microscopy, Oberkochen, Germany) with objective 40× magnification.

### 4.13. RT-qPCR

Crude hexane extract, citronellol, citronellal, or doxorubicin were treated on MDA-MB-231 cells for 24 h. Cells treated with culture medium only were used as vehicle control. Total RNA was extracted from MDA-MB-231 cells using Ribozol^TM^ RNA extraction reagent kits (AMRESCO, VWR Life Science, OH, USA). Briefly, cDNA synthesis was performed using Tetro cDNA synthesis kits (Bioline Reagents Ltd., London, UK). Real-time PCR was carried out using SensiFAST^TM^ SYBR No-ROX kits (Bioline Reagents Ltd., London, UK). The initial denaturing temperature started from 95 °C at 1 min, with 45 cycles of 15 s denaturation followed by 30 s of annealing and elongation at 60 °C. The whole reaction was performed in a CFX96 Touch Real-Time PCR Detection System (Bio-Rad Laboratories, Hercules, CA, USA). Primer sequences of β-actin, Bcl-2, and Bax were obtained from previous studies and checked by the BLAST tool from the National Institute of Health (Table 2). All samples were performed in triplicate and levels of gene expression were normalized to the level of β-actin as the reference gene. The relative amount of target gene expression was calculated using 2^−ΔΔCT^ [29].

### 4.14. Western Blot Analysis

Crude hexane extract, citronellol, citronellal, or doxorubicin were treated on MDA-MB-231 cells for 24 h. Cells treated with culture medium only were used as vehicle control. RIPA lysis buffer (Sigma-Aldrich, Inc.) containing 1% of protease and phosphatase inhibitor cocktail (Sigma-Aldrich, Inc.) was used to extract total protein. Proteins from cell lysates were quantified using Pierce™ Coomassie (Bradford) protein assay kit (Thermo Fisher Scientific, Inc., Waltham, MA, USA) by measuring absorbance at 595 nm. An aliquot of 50 µg of total protein from cell lysates was introduced to 12% SDS-PAGE and then transferred to PDVF membranes. Five percent skim milk in TBST was used as blocking buffer for 1 h at room temperature. Primary antibody against proteins β-actin, Bcl-2, Bax, pro-caspase-3, and cleaved-caspase-3 from (Santa Cruz Biotechnology, Inc., Dallas, TX, USA) were probed on membranes (1:1000 dilution) and shaken overnight at 4 °C. After washing three times for 5 min each with TBST, the membranes were incubated with HRP-conjugated anti-mouse IgG (Santa Cruz Biotechnology) in 1% BSA-TBST (1:10,000 dilution) at room temperature for 1 h, followed by washing three times. Protein bands were detected by adding horse radish peroxidase chemiluminescence substrate and quantified by Chemi Doc XRS Imaging System (Bio-Rad Laboratories, Inc., Hercules, CA, USA).

### 4.15. Data Analysis

All the experiments were performed in triplicate and data were expressed as means ± SEM. One-way ANOVA with Dunnett’s test was used to analyze degree of significance between controls and samples. *p* value < 0.05 was considered significant.

## 5. Conclusions

**Figure 9:**
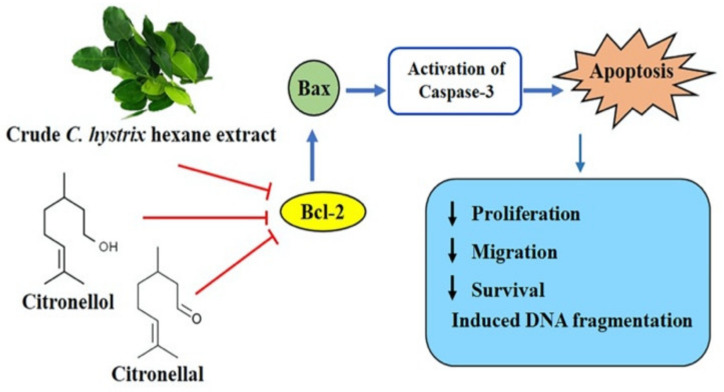
Inhibition of Bcl-2 protein in MDA-MB-231 cells by *C. hystrix*, citronellal, and citronellol leading to activation of caspase dependent apoptotic pathway.

Our results demonstrated that crude *C. hystrix* hexane extract and its compounds citronellol and citronellal showed anticancer effects by inducing apoptosis in the triple negative breast cancer MDA-MB-231 cell line through inhibition of the anti-apoptotic protein Bcl-2, leading to activation of the pro-apoptotic Bax protein and inducing the downstream caspase-dependent apoptosis pathway by activating caspase-3 protein. Crude hexane extract, citronellol, and citronellal downregulated the active state of the cells and this decreased their proliferation rate and ability to migrate and survive under harsh environmental conditions (Figure 9).

## Figures and Tables

**Table 1:** The identified compounds from crude hexane by GC-MS.

No.	RT (min)	R.I ^1^	Identified Compounds	Classification	R.A ^2^ (%)
1	9.23	1100	Linalool	Monoterpene ^a^	0.34
2	10.56	1146	Isopulegol	Monoterpene ^a^	1.01
3	10.75	1154	Citronellal	Monoterpene ^a^	0.67
4	11.46	1179	Terpinen-4-ol	Monoterpene ^a^	0.29
5	11.83	1193	α-Terpineol	Monoterpene ^a^	0.11
6	12.85	1229	Citronellol	Monoterpene ^a^	1.42
7	14.08	1354	3,7-dimethyloct-1,7-dien-3,6-diol	Monoterpene ^a^	0.15
8	16.23	1354	α-Cubebene	Sesquiterpene ^b^	0.94
9	16.94	1381	α-Copaene	Sesquiterpene ^b^	1.44
10	17.29	1395	β-Cubebene	Sesquiterpene ^b^	0.34
11	18.08	1426	Caryophyllene	Sesquiterpene ^b^	1.59
12	18.30	1435	Bicyclosequiphellandrene	Sesquiterpene ^b^	0.20
13	18.93	1460	α-Humulene	Sesquiterpene ^b^	0.23
14	19.47	1482	γ-Muurolene	Sesquiterpene ^b^	0.12
15	20.05	1505	α-Muurolene	Sesquiterpene ^b^	0.31
16	20.61	1529	δ-Cadinene	Sesquiterpene ^b^	0.62
17	21.23	1555	Elemol	Sesquiterpene ^a^	0.13
18	21.53	1568	Nerolidol	Sesquiterpene ^a^	0.71
19	21.98	1586	Spathulenol	Sesquiterpene ^a^	1.34
20	22.12	1592	Caryophyllene oxide	Sesquiterpene ^a^	3.74
21	23.21	1641	Caryophylladienol	Sesquiterpene ^a^	0.39
22	23.67	1661	Viridiflorene	Sesquiterpene ^b^	0.20
23	24.05	1678	Caryophyllenol	Sesquiterpene ^a^	1.14
24	25.90	1764	Tetradecanoic acid	Fatty acid ^a^	0.34
25	26.29	1785	Alloaromadendrene oxide	Sesquiterpene ^a^	0.22
26	27.63	1847	Hexahydrofarnesyl acetone	Sesquiterpene derivative	0.81
27	29.24	1927	Methyl palmitate	Fatty acid ^a^	0.30
28	30.19	1976	Palmitic acid	Fatty acid ^a^	6.82
29	32.79	2015	Phytol	Diterpene	0.40
30	33.30	2144	Linoleic acid	Fatty acid ^a^	1.89
31	33.40	2149	(6*Z*),(9*Z*)-Pentadecadien-1-ol	Fatty acid ^a^	2.39
32	44.09	2833	Supraene	Triterpene	0.31
33	44.92	2893	cis-2,6-Dimethyl-2,6-octadiene	Monoterpene ^b^	2.19
34	48.24	3103	Tetracosane	Hydrocarbon	3.21
35	48.98	3139	α-Tocopherol	Vitamin	0.56
36	49.15	3147	Pentacosane	Hydrocarbon	1.03
37	50.92	3227	Campesterol	Phytosterol	0.46
38	51.73	3260	Stigmasterol	Phytosterol	1.07
39	52.86	3309	Heneicosane	Hydrocarbon	2.57
40	53.00	3350	1-Eicosanol	Fatty alcohol	0.37
41	53.27	3317	γ-Sitosterol	Phytosterol	2.90
42	53.81	3335	Lanosterol	Triterpene	2.45
43	58.99	3485	Lupenyl acetate	Triterpene	0.68
44	60.02	3510	17-Pentriacontene	Hydrocarbon	2.23
45	62.13	3556	Neophytadiene	Diterpene ^b^	0.61
Total R.A of identified compounds					51.24%
Oxygenated monoterpenes					3.99%
Hydrocarbon monoterpene					2.19%
Oxygenated sesquiterpenes					7.67%
Hydrocarbon sesquiterpenes					5.99%
Hydrocarbons					9.04%
Fatty acids and fatty alcohols					12.11%
Other					10.25%

^1^ Retention indices (R.I) were calculated using a homologous series of n-alkanes (C8–C32); ^2^ Relative amounts (R.A) were obtained by peak areas normalization; ^a^ Oxygenated form; ^b^ Hydrocarbon form.

**Table 2:** Primer sequences.

Target Genes	Primer Sequences	References
*β-actin*	Fw: 5′-AGAAAATCTGGCACCACACC-3′	[30]
	Rw: 5′-CCATCTCTTGCTCGAAGTCC-3′	
*Bcl-2*	Fw: 5′-GATGTGATGCCTCTGCGAAG-3′	[31]
	Rw: 5′-CATGCTGATGTCTCTGGAATCT-3′	
*Bax*	Fw: 5′-GGTTGTCGCCCTTTTCTA-3′	[31]
	Rw: 5′-CGGAGGAAGTCCAATGTC-3′	

## Supplementary Materials

The following are available online at https://www.mdpi.com/1424-8247/13/12/476/s1, Figure S1: Total ion chromatogram of crude hexane extract, Figure S2: IC50 value of treatments on human monocyte derived macrophages, Figure S3: Full length of Western blot analysis, Figure S4: TLC fingerprints of crude *C. hystrix* hexane extract, ethyl acetate extract, ethanolic extract, and two standard compounds (citronellol and citronellal).

## Abbreviations

*C. hystrix*	*Citrus hystrix* DC.
ER	Estrogen receptor
GC-MS	Gas chromatography-Mass spectrometry
Her-2	Human epidermal receptor 2
PR	Progesterone receptor
RA	Retention areas
RI	Retention indices
RT	Retention times
TNBC	Triple Negative Breast Cancer
