# Supplementary material for: Anticancer Effect of Citrus hystrix DC. Leaf Extract and Its Bioactive Constituents Citronellol and, Citronellal on the Triple Negative Breast Cancer MDA-MB-231 Cell Line
Source: Pharmaceuticals (Basel). 2020 Dec 18;13(12):476. doi: 10.3390/ph13120476 (PMC7766836; doi:10.3390/ph13120476)
Supplement: Supplementary file 1 [file pharmaceuticals-13-00476-s001.pdf]

Supplement Figures

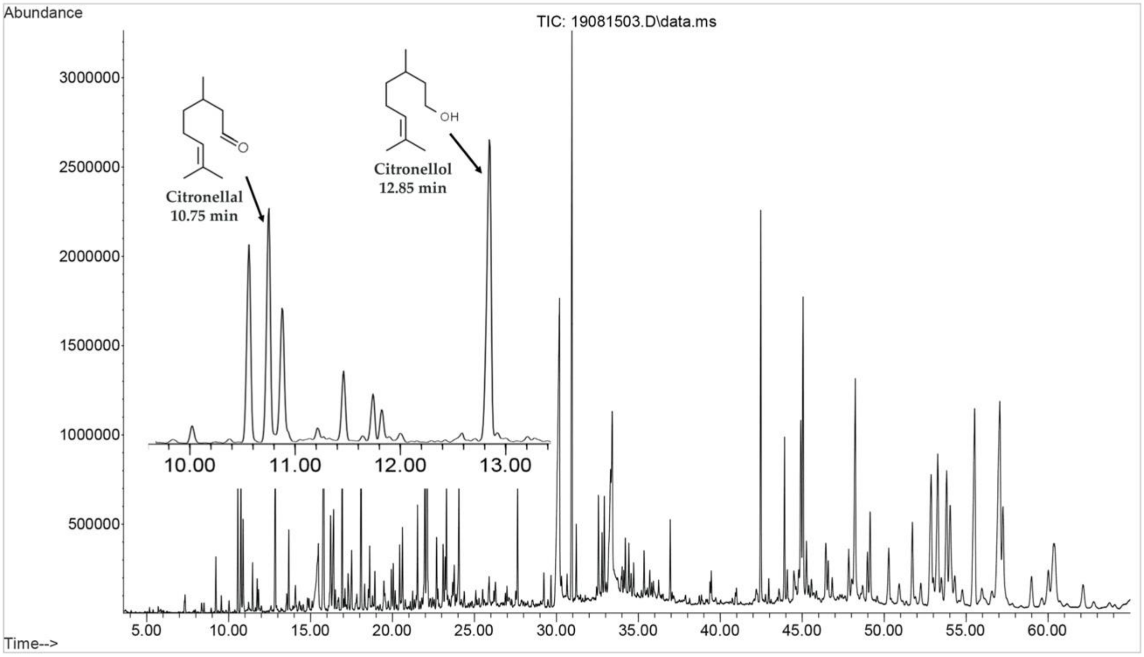

Supplementary Figure S1 Total ion chromatogram of crude hexane extract (50 mg/ml) was obtained from GC-MS analysis. Two peaks of citronellal and citronellol were observed at retention times at 10.75 min and 12.85 min.

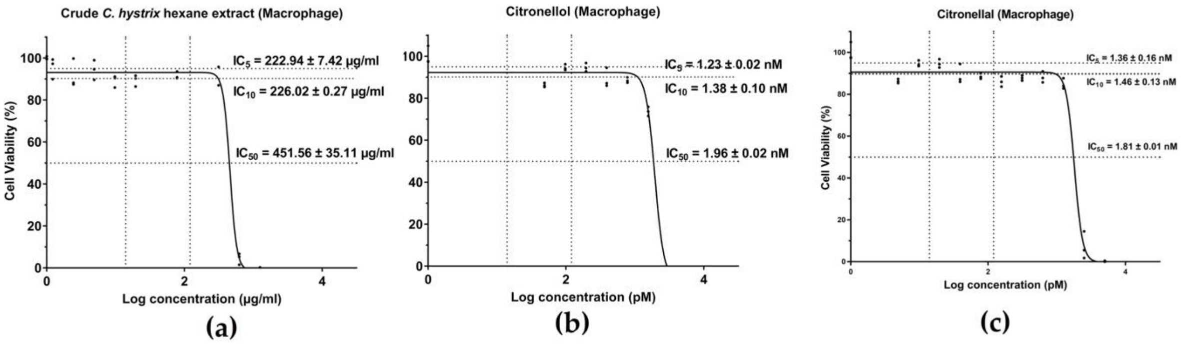

Supplementary Figure S2 IC_{50} value of treatments on human monocyte derived macrophages: (a) crude hexane extract, (b) citronellol and (c) citronellal.

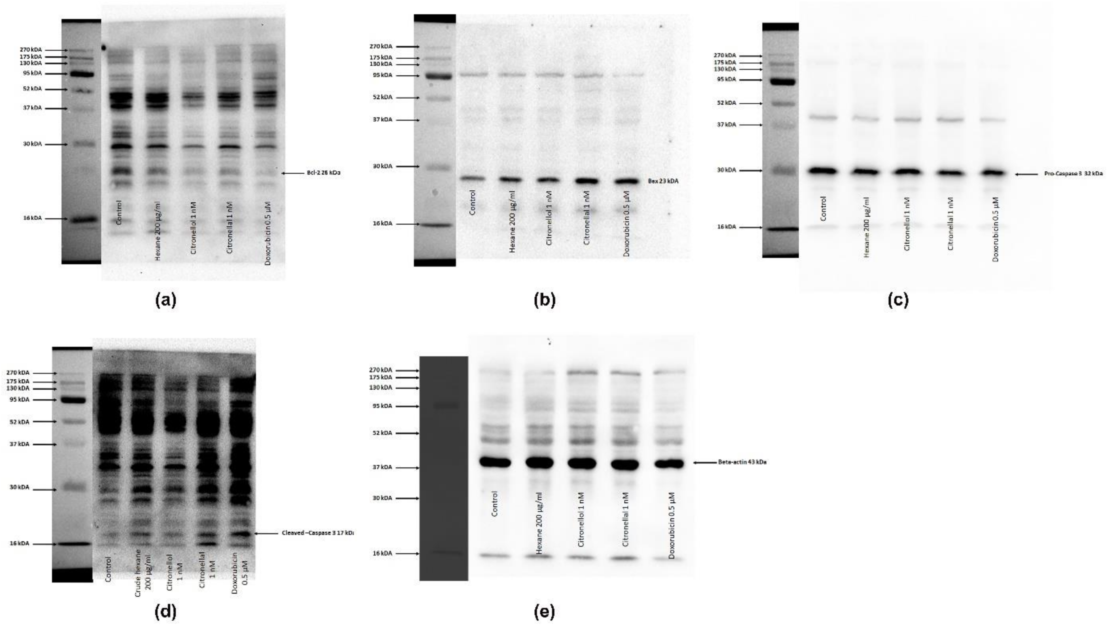

Supplementary Figure S3 Full length of Western blot analysis (a) Bcl-2, (b) Bax, (c) Pro-caspase-3, (d) Caspase-3 and (e) beta actin

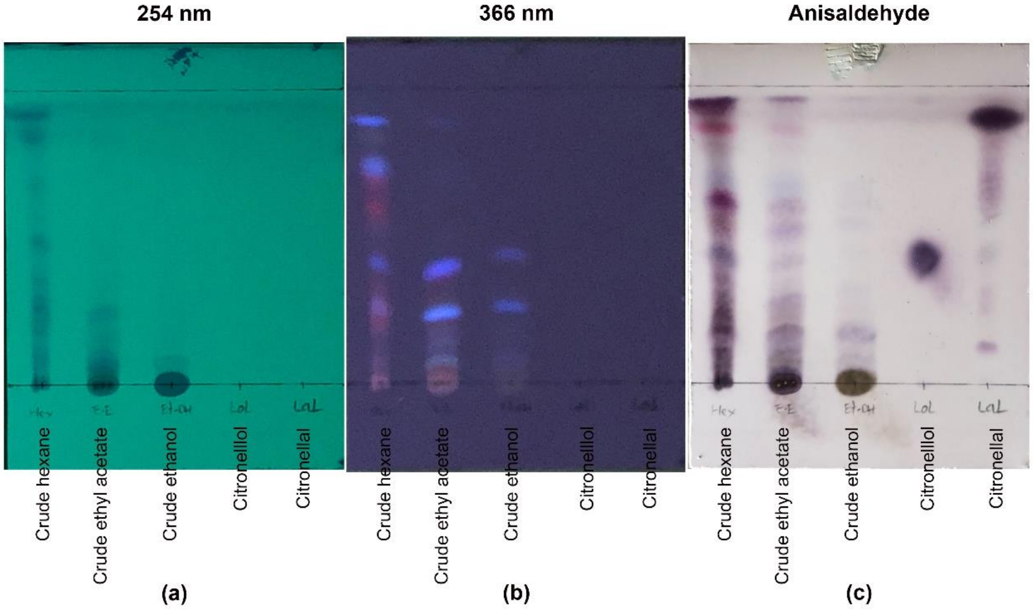

Supplementary Figure S4. TLC fingerprints of crude *C. hystrix* hexane extract, ethyl acetate extract, ethanolic extract, and two standard compounds (citronellol and citronellal) using hexane:ethyl acetate (7:3 v/v) as mobile phase . The plates were visualized without coloration under (a) 254 nm UV light, (b) 366 nm UV light and (c) after coloration with anisaldehyde-sulfuric acid reagent.
